# Post-Traumatic Stress Symptoms and Dissociation in Emergency Medical Service Personnel

**DOI:** 10.3390/healthcare14162534

**Published:** 2026-08-13

**Authors:** Rinat Tsruya, Eran Shadach, Leah Shelef

**Affiliations:** 1 School of Social Sciences, The Academic College of Tel Aviv-Yaffo, Tel Aviv Yaffo 6818211, Israel; rinatts@mta.ac.il; 2 School of Social Work, Sapir College, Ashkelon 7916500, Israel; lshelef4@gmail.com

**Keywords:** post-traumatic stress symptoms, depression, dissociation, trauma, emergency medical services

## Abstract

**Background:** Emergency medical service (EMS) personnel and volunteers are frequently exposed to traumatic events, placing them at elevated risk for psychological distress, including post-traumatic stress symptoms (PTSSs), depression, and dissociation. This study examined the relationships among PTSS, depression, and dissociation in Israeli EMS personnel, with a particular focus on whether dissociation mediates the association between depression and PTSS. **Methods:** A cross-sectional study was conducted with 60 EMS personnel in Israel, including 7 paid employees, 37 volunteers, and 16 participants who did not report their employment status. PTSSs were assessed using the Impact of Event Scale-Revised (IES-R), depressive symptoms were measured with the Depression, Anxiety, and Stress Scale (DASS-21), and dissociative experiences were evaluated using the Dissociative Experiences Scale-II (DES-II). Correlational analyses and mediation analyses were performed. **Results:** Significant positive correlations were found among PTSS, depression, and dissociation. However, mediation analysis indicated that dissociation did not significantly mediate the relationship between depression and PTSS. **Conclusions:** Although depression and dissociation were both strongly associated with PTSS, dissociation did not account for the association between depression and PTSS in this sample of EMS personnel. These findings suggest that other psychological mechanisms may underlie the relationship between depression and trauma-related symptoms in emergency responders. Further research is warranted to identify these mechanisms and to inform targeted interventions aimed at promoting the mental health and resilience of EMS personnel.

## 1. Introduction

While many individuals exposed to trauma demonstrate resilience, a substantial minority develop post-traumatic stress disorder (PTSD), the primary psychiatric disorder associated with traumatic experiences [1]. According to the Diagnostic and Statistical Manual of Mental Disorders (DSM-5-TR), PTSD may develop following direct exposure to a traumatic event, witnessing such an event, or repeated exposure to aversive details of traumatic experiences [2].

Epidemiological studies estimate that PTSD affects approximately 3–5% of the general population each year [1], although the risk of developing PTSD following trauma varies considerably depending on the type and severity of exposure [3]. PTSD is characterized by four symptom clusters: intrusion, avoidance, negative alterations in cognition and mood, and alterations in arousal and reactivity [2]. Individuals with PTSD commonly experience intrusive memories, recurrent nightmares, hyperarousal, impaired concentration, sleep disturbances, emotional numbing, avoidance of trauma-related reminders, and social withdrawal. Beyond its psychological burden, PTSD has been associated with impaired occupational functioning, increased healthcare utilization, and psychiatric comorbidities such as depression and anxiety [2,4].

Although PTSD occurs across a wide range of populations, certain occupational groups are particularly vulnerable because of repeated exposure to traumatic events. Law enforcement officers, firefighters, and emergency medical service (EMS) personnel routinely encounter severe injuries, death, and human suffering [5]. Consequently, EMS personnel are repeatedly exposed to traumatic stressors as part of their routine duties [6], placing them at substantially greater risk of developing PTSD and related psychological disorders than the general population [7,8,9]. As a result, EMS personnel have received increasing attention as a population at elevated risk for trauma-related psychopathology [10,11].

### 1.1. PTSD in Emergency Personnel

Research consistently demonstrates that EMS personnel are at increased risk of PTSD. Unlike the single-incident trauma more commonly experienced in the general population, EMS personnel are repeatedly exposed to cumulative stressors, including severe injuries, resuscitation attempts, fatal accidents, and death notifications [10,12]. Such repeated exposure has been associated with persistent psychological distress and trauma-related symptoms [10].

PTSD prevalence rates among emergency personnel range from approximately 10% to 20%, substantially exceeding the 1–5% prevalence observed in the general population [7,8]. Ambulance personnel appear to be particularly vulnerable, even compared with other emergency responders [12]. Although repeated trauma exposure is a major risk factor [7,8], protective factors such as careful personnel selection, professional training, and strong team cohesion may reduce vulnerability, contributing to the so-called “healthy worker effect” [5]. Nevertheless, cumulative trauma exposure has been associated with increased prevalence and severity of PTSD, reduced spontaneous recovery, and higher rates of depression and anxiety among EMS personnel [6,13,14].

### 1.2. PTSD and Depression

Depression is one of the most common psychiatric conditions accompanying PTSD and represents one of its most clinically significant comorbidities [15,16,17,18,19]. Major depressive disorder (MDD) is characterized by persistent depressed mood, diminished interest or pleasure, feelings of worthlessness or excessive guilt, fatigue, impaired concentration, and other cognitive and somatic symptoms that substantially impair daily functioning [2].

Approximately 30–50% of individuals with PTSD also meet diagnostic criteria for MDD [15,20]. Depression and PTSD share substantial overlap in both symptom presentation and underlying psychological mechanisms. Depressive symptoms, including hopelessness, anhedonia, and negative self-perceptions, may exacerbate PTSD severity and contribute to poorer functional outcomes [21]. Individuals with co-occurring PTSD and MDD generally experience more severe psychopathology and greater functional impairment than those with either disorder alone [22].

Beyond its high prevalence, depression may influence the way traumatic memories are encoded, processed, and integrated, thereby affecting emotional, cognitive, and physiological responses following trauma exposure [23]. It has also been proposed that depression interacts with trauma-related processes, including dissociation, that contribute to the development and maintenance of PTSS [24]. Understanding these relationships may therefore clarify the mechanisms linking depression and PTSD.

### 1.3. Dissociation as a Mediating Factor

Dissociation is widely regarded as a protective psychological response to overwhelming experiences through temporarily disrupting the integration of consciousness, memory, emotion, and identity [25,26]. Although dissociation may reduce immediate distress during a traumatic event, it may also manifest as persistent depersonalization and derealization, that is, a sense of detachment from oneself or the surrounding environment [2]. Consistent with this view, persistent dissociative responses have been associated with impaired processing of traumatic memories, avoidance of trauma-related cues, and greater severity and chronicity of PTSS [27,28].

From this perspective, dissociation may initially function as an adaptive emotion-regulation strategy by reducing awareness of overwhelming emotional experiences [29]. However, when maintained after the traumatic event, it may interfere with emotional processing and hinder recovery. Individuals exposed to traumatic or highly stressful events frequently report both depressive and dissociative symptoms [30,31]. Previous studies have demonstrated significant associations among dissociation, depression, and PTSD, highlighting the clinical importance of examining these interrelated processes [31,32,33].

Dissociation is a multidimensional construct encompassing a range of experiences that vary in severity and clinical significance. It may refer to structural dissociation of the personality, peritraumatic dissociation occurring during or immediately after trauma, or persistent dissociative symptoms following trauma. The present study focuses on dissociative experiences as assessed by the Dissociative Experiences Scale-II (DES-II), which measures self-reported experiences ranging from relatively common phenomena, such as absorption and lapses in awareness, to more clinically significant experiences, including depersonalization, derealization, and dissociative amnesia.

Given the high frequency of traumatic exposure and the substantial emotional demands of emergency work, EMS personnel provide an important population in which to examine these relationships. Previous research has documented elevated rates of depression and dissociative symptoms among emergency responders, often associated with chronic occupational stress and repeated trauma exposure [10,12]. Within this context, prolonged dissociation may represent a mechanism through which depressive symptoms contribute to the development or maintenance of PTSS. Examining this possibility may improve understanding of trauma-related psychopathology in EMS personnel and help identify targets for psychological intervention.

### 1.4. The Present Study

Although PTSD among EMS personnel has been extensively investigated [6,8,10,12,14,34], considerably less attention has been given to the combined roles of depression and dissociation in trauma-related psychopathology. Previous research suggests that dissociation may function as a psychological mechanism linking depression and PTSD [24]. Because dissociation can impede the integration and processing of traumatic experiences while being associated with both depressive symptoms and PTSS severity, it may partially mediate the relationship between depression and PTSS. From this perspective, depressive symptoms may be associated with greater reliance on dissociative coping, which may, in turn, be associated with more severe post-traumatic stress symptoms, making dissociation a plausible mediator of the association between depression and PTSS.

It should be emphasized that, throughout this study, the term dissociation refers specifically to self-reported dissociative experiences measured by the Dissociative Experiences Scale-II (DES-II) and should not be interpreted as assessing structural dissociation of the personality or persistent pathological dissociation. Moreover, the present study is an exploratory cross-sectional study that tests a statistical indirect-effect model involving dissociation rather than demonstrating a psychological mechanism underlying the observed associations. Nevertheless, examining dissociative experiences alongside post-traumatic stress and depressive symptoms may contribute to a more comprehensive understanding of psychological responses to repeated occupational trauma and may help inform future screening and intervention strategies.

Subject to these limitations, the present study tested the following hypotheses regarding the associations among the study variables and a statistical indirect-effect model involving dissociation:

1. PTSS would be positively associated with depressive symptoms.
2. PTSS would be positively associated with dissociation.
3. Depression would be positively associated with dissociation.
4. Dissociation would statistically mediate (i.e., serve as an indirect effect in the tested model) the relationship between depression and PTSS.

## 2. Method

### 2.1. Participants

A cross-sectional study was conducted with 60 EMS personnel between the ages of 18 and 67 (58.3% male, 41.7% female) in Israel. They included 7 paid employees, 37 volunteers, and 16 participants who did not report their employment status. They were recruited through social media forums and through in-person invitations disseminated at their workplaces. Because of this, the exact number of personnel who received it could not be determined, although it is estimated that between 100 and 120 individuals were exposed to the invitation. All participants were fluent in Hebrew and worked or volunteered in emergency medical services (EMS), primarily as medics and paramedics in the two major national emergency services in Israel, Magen David Adom (MDA) and United Hatzalah (UH). The exclusion criteria consisted of (a) individuals who were on leave or not actively serving as first responders at the time of data collection and (b) personnel who were not fluent in Hebrew.

### 2.2. Measures

Demographic questionnaire: The questionnaire included questions relating to age, gender, place of residence, work experience, external stress exposure, and length of service. External stress exposure was assessed using a single self-report item asking participants whether they had experienced a traumatic event outside the scope of their emergency service duties. Responses were recorded as Yes or No and coded as a binary variable (1 = external traumatic exposure, 0 = no external traumatic exposure). Because the purpose of this variable was to account for participants’ perceived exposure to traumatic experiences outside their occupational role, classification was based on participants’ self-report rather than on a formal clinical assessment. This variable was included as a covariate in the regression analyses to control for the potential influence of non-occupational traumatic exposure on the study outcomes. The purpose of the questionnaire was to provide a general background of the participants and their suitability for this study.

The Impact of Event Scale-Revised (IES-R [35], translated into Hebrew [36]): This is a 22-item self-report questionnaire adapted from the original IES questionnaire [37]. The IES-R consists of seven additional items to encompass the DSM criteria for PTSS. This measure is designed to detect an individual’s degree of subjective distress in response to a traumatic experience and subsequently indicates the level of impairment from post-traumatic stress.

The questionnaire comprises three subscales: intrusion, avoidance, and hyperarousal. The intrusion subscale encompasses intrusive thoughts, feelings, and imagery (e.g., “I thought about it when I didn’t mean to”), as well as nightmares and dissociative-like re-experiencing (e.g., “I found myself acting or feeling like I was back that time”). This subscale includes 8 items: 1, 2, 3, 6, 9, 14, 16, and 20. The avoidance subscale covers numbness (e.g., “My feelings about it were kind of numb”) and avoidance of feelings, situations, and ideas (e.g., “I avoided letting myself get upset when I thought about it or was reminded of it”). This subscale also includes 8 items: 5, 7, 8, 11, 12, 13, 17, and 22. The hyperarousal subscale encompasses anger, irritability, hypervigilance (e.g., “I felt watchful and on-guard”), and difficulty concentrating. It includes 6 items: 4, 10, 15, 18, 19, and 21. The scoring method is a 5-point Likert scale, ranging from 0 (“Not at all”) to 4 (“Extremely”). The IES-R results consist of separate scores for all three subscales and a total score. Total scores are divided into 0–23 (normal), 24–32 (mild psychological impact), 33–36 (moderate psychological impact), and 37 and above (severe psychological impact). The IES-R was found to have excellent internal consistency in multiple studies, with Cronbach’s alpha values ranging between 0.85 and 0.95 [38,39]. The internal consistency in the current study for the intrusion scale was *α* = 0.90, for the avoidance scale *α* = 0.87, and for the hyperarousal scale *α* = 85.

The Depression Anxiety Stress Scale (DASS-21 [40], translated into Hebrew [41]): This is a self-report questionnaire adapted as a short form of the original 42-item DASS, consisting of 21 items that assess overall psychological distress while also measuring three symptom subscales: depression, anxiety, and stress. The depression subscale evaluates dysphoria, hopelessness, devaluation of life, self-deprecation, and anhedonia. An example item is “I felt that life was meaningless”. This scale includes items 3, 5, 10, 13, 16, 17, and 21. The anxiety subscale assesses autonomic arousal typical of anxiety, such as trembling, sweating, feelings of panic, and the fear of losing control. The anxiety items are designed to assess the respondent’s physical symptoms of anxious arousal, rather than the persistent worry characteristic of generalized anxiety disorder. An example item is “I was worried about situations in which I might panic and make a fool of myself”. This scale includes items 2, 4, 7, 9, 15, 19, and 20. The stress subscale evaluates levels of chronic arousal, difficulty relaxing, nervous arousal, agitation, and impatience. The stress items assess the respondent’s level of tension and ongoing arousal, reflecting how strongly they feel burdened or overwhelmed by everyday stress. An example item is “I found it difficult to relax”. The stress subscale includes items 1, 6, 8, 11, 12, 14, and 18. Items in the DASS-21 questionnaire are rated on a 4-point Likert scale ranging from 0 (“Did not apply to me at all”) to 3 (“Applied to me very much or most of the time”). The questionnaire provides a total score (ranging from 0 to 63) as well as separate scores for each subscale. Raw scores for each DASS-21 subscale range from 0 to 21. To enable interpretation according to the conventional DASS severity classifications based on the original 42-item DASS, scores for each subscale were multiplied by two, yielding a range of 0 to 42 for each subscale. The doubled subscale scores were used consistently in all statistical analyses, categorizations, and severity classification. In the present study, analyses primarily focused on the DASS-21 depression subscale, given its relevance to the study’s research questions and mediation model. Scores for each subscale are categorized into five severity ranges: normal, mild, moderate, severe, and extremely severe. The overall scale demonstrates strong internal reliability with a coefficient of 0.93. The reliability coefficients for the subscales are 0.88 for depression, 0.82 for anxiety, and 0.90 for stress [42]. The internal consistency of the depression subscale in the current study was *α* = 0.88, for anxiety *α* = 0.87, and for stress *α* = 0.90.

The Dissociative Experiences Scale-II (DES-II [43,44]; translated into Hebrew [45]): This is a 28-item, self-report measure of dissociative experiences. The scale examines an assortment of dissociative experiences, ranging from relatively normal experiences like daydreaming to complex experiences. The scale is of particular use in measuring dissociation among people with PTSS. The DES-II examines three main domains of dissociative experiences: amnesia, depersonalization, and absorption. The amnesia subscale assesses gaps in memory and awareness (e.g., “Some people have the experience of finding themselves in a place and have no idea how they got there”). This scale includes 6 items: 3, 4, 5, 8, 25, and 26. The depersonalization subscale measures feelings of detachment and unreality in relation to the surroundings or to oneself (e.g., “Some people have the experience of feeling that other people, objects, and the world around them are not real”). This scale contains 6 items: 7, 11, 12, 13, 27, and 28. The absorption subscale measures a state of deep absorption in an activity, characterized by a limited perception of external stimuli (e.g., “Some people find that they become so involved in a fantasy or daydream that it feels as though it were really happening to them”). This scale also includes 6 items: 2, 14, 15, 17, 18, and 20. The items in the DES-II are rated on a scale ranging from 0% (Never) to 100% (Always). The DES-II yields both overall and subscale scores by averaging responses across 28 items. Elevated scores reflect a higher frequency and intensity of dissociative experiences. When the DES-II score exceeds 30%, further assessment for a dissociative disorder is recommended. The DES-II shows excellent internal consistency, with Cronbach’s alpha values ranging from 0.93 to 0.95 across multiple studies [44,46,47]. The internal consistency in the current study for the amnesia scale was *α* = 0.85, the depersonalization scale *α* = 0.84, and the absorption scale *α* = 0.83.

### 2.3. Procedure

Participants were approached during an unprecedented national crisis via MDA and UH social media platforms, as well as directly in EMS stations in the central cities of Jerusalem, Ramat Gan, and Tel Aviv. The call was directed at medics and paramedics in active service who were working or volunteering in EMS positions, and the questionnaires were completed via a Google Forms online platform. All participants who entered the study completed it successfully and were included in the final analysis. Upon logging into the online platform, participants first read and signed an informed consent form. Then, they completed a demographic questionnaire, followed by three standardized self-report measures: the Impact of Event Scale-Revised (IES-R); the Dissociative Experiences Scale-II (DES-II); and the Depression, Anxiety, and Stress Scale (DASS-21).

### 2.4. Statistical Analysis

Data were analyzed using IBM SPSS version 29. Descriptive statistics were calculated to present participants’ demographic characteristics and the main study variables of PTSS, depression, and dissociation, including means and standard deviations for continuous variables and frequencies and percentages for categorical variables. Due to the nonparametric nature of the data, Spearman’s correlation coefficients were used to examine the associations among PTSS, depression, and dissociation. Mann–Whitney U tests were employed to assess potential differences in post-traumatic, depressive, and dissociative symptom levels across background variables (e.g., gender, district).

Finally, to test the fourth hypothesis, a mediation analysis was performed using PROCESS Model 4 [48], which relies on bootstrapped confidence intervals that are considered robust to violations of normality assumptions. Indirect associations were estimated using 5000 bootstrap samples and bias-corrected 95% confidence intervals. Bootstrapping procedures are considered relatively robust to violations of normality assumptions and are appropriate for mediation analyses in modest sample sizes. Regression assumptions and influence diagnostics were examined prior to analysis, and no substantial violations or influential outliers were identified. This analysis aimed to examine whether dissociation mediated the relationship between depression and PTSS. Depression was specified as the independent variable (X), dissociation as the mediator (M), and PTSS as the dependent variable (Y). To account for potential confounding influences, gender, age, seniority, and external stress exposure were included as covariates in the mediation analyses. Unstandardized regression coefficients (b) were estimated.

Given the number of correlational analyses conducted, false discovery rate (FDR) correction using the Benjamini–Hochberg procedure [49] was applied to the resulting *p*-values (All significance indicators refer to FDR-adjusted values). A sensitivity power analysis conducted using G*Power (version 3.1.9.7) indicated that with N = 60, α = 0.05, and power = 0.80, the study was sufficiently powered to detect associations of approximately f^2^ = 0.25 or larger in the regression analyses.

## 3. Results

Demographic characteristics of the participants are presented in Table 1. The participants were primarily from the Jerusalem district (41.6%), the central district (25%), and the northern district (23.3%). Of the participants, 61.6% reported recent exposure to a traumatic incident outside of work. The seniority in their positions ranged from several months to 30 years, with a mean of 4.94 years (SD = 5.2). The number of shifts participants reported completing each month ranged from 1 to 28 shifts, averaging 10.7 shifts per month (SD = 8.6).

The study variables are presented in Table 2. Among the participants, 25% were categorized with severe PTSS, 5% with moderate symptoms, and 11.66% with mild symptoms based on the IES-R questionnaire. In the depression scale, 11.66% reported severe or extremely severe symptoms, whereas 15% reported moderate symptoms, and 13.33% reported mild symptoms. In addition, 30% of participants scored above the commonly used DES-II screening cut-off of 30, indicating that further clinical assessment may be warranted (DES > 30%). Regarding the mean scores, the general PTSS mean was in the mild range, amounting to 24.96 (SD = 18.74). Within the depression scale, however, the overall mean was within the normal range (M = 8.5, SD = 10.01). The average level of dissociation in the sample was below the threshold index, with a mean of 25.97% (SD = 14.08).

The interaction between the background variables and the main variables is presented in Table 3. For demographic and service-related variables and their relation to PTSS, depression, and dissociation, only gender and previous exposure to traumatic events showed significance in association with the depression scale. Women demonstrated significantly higher depression symptoms compared to men (*p* = 0.034). Similarly, previous exposure to a traumatic event increased the level of depression (*p* = 0.022).

The first three hypotheses were supported, as there was a positive relationship between PTSS and depression symptoms, as well as between PTSS and dissociation symptoms and between depression and dissociation symptoms. As can be seen in Table 4, Spearman’s correlation demonstrates a moderate positive association between the three variables: PTSS (i.e., IES-R), depression (i.e., depression scale of DASS-21), and dissociation (i.e., DES-II). Accordingly, a higher level of depression was correlated with a higher level of PTSS (*r* = 0.519; *p* < 0.01), and so was a higher level of dissociation correlated with a higher level of PTSS (*r* = 0.536; *p* < 0.01). Additionally, depression and dissociation were positively correlated (*r* = 0.512; *p* < 0.01). Table 4 presents the correlations between the total scores of the three questionnaires and includes all correlations among their respective subscale scores.

**Figure 1:**
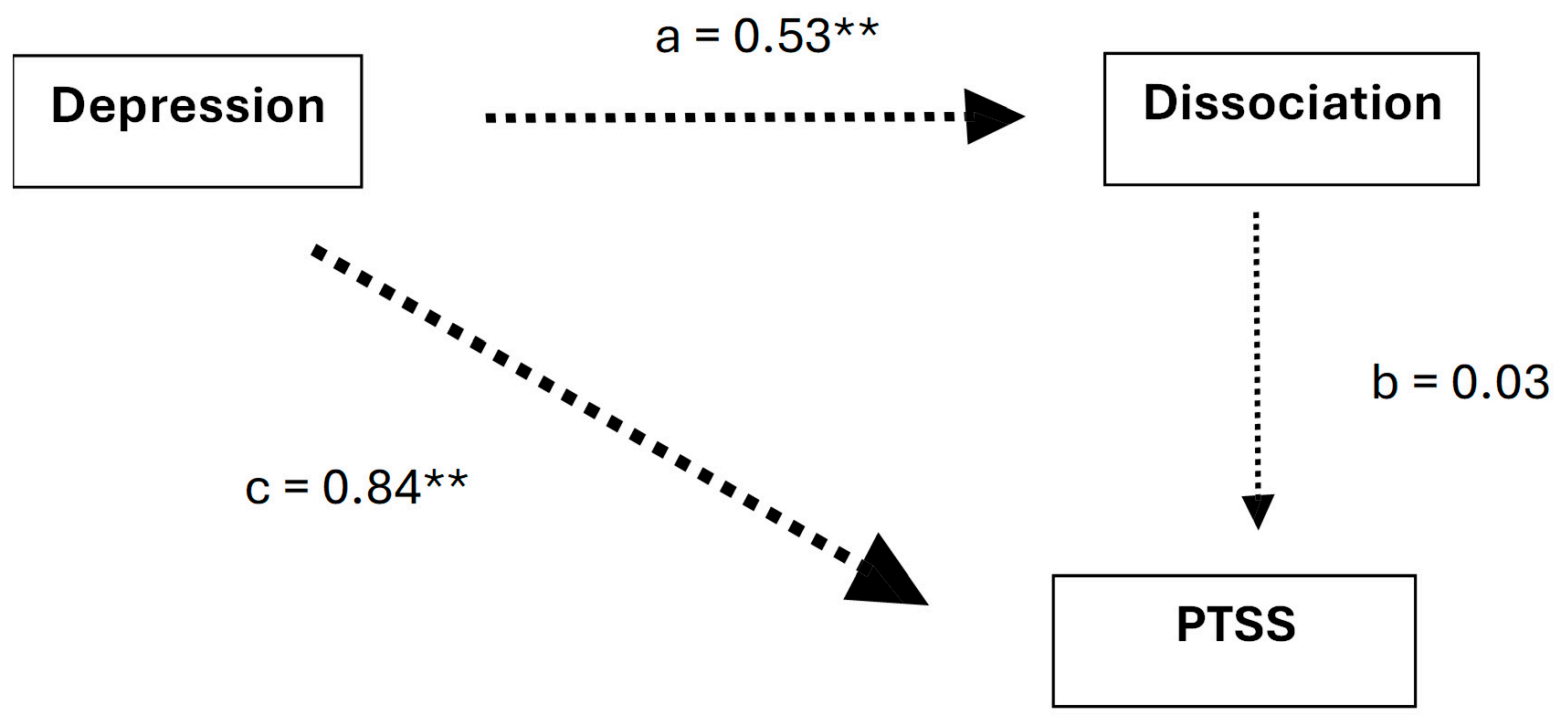
Path model between dissociation, depression, and PTSS. ** Regression coefficient is significant at the 0.01 level (2-tailed).

**Figure 2:**
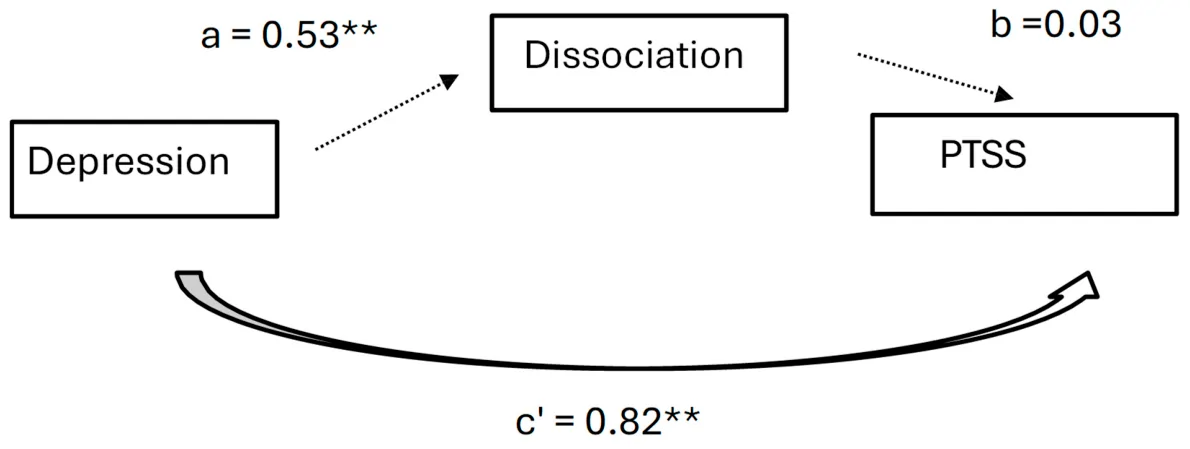
Mediation model with dissociation as a mediator between depression and PTSS. ** Regression coefficient is significant at the 0.01 level (2-tailed); indirect effect (a × b) = 0.01; 95% BCa bootstrap CI [−0.14, 0.36]; not significant.

Table 5 presents standardized (β) and unstandardized (B) coefficients; figures display the unstandardized coefficients. DES-II scores were entered into the mediation model on the 0–100 metric, consistent with the descriptive statistics. The model was estimated while controlling for gender, age, seniority, and external stress exposure (N = 60; percentile bootstrap with 5000 samples, seed = 12,345). As can be seen in Table 5 and Figure 1, depression significantly predicted higher levels of dissociation (B = 0.53, SE = 0.18, *p* = 0.004, 95% CI [0.17, 0.90]). Dissociation, in turn, did not significantly predict PTSS (B = 0.03, SE = 0.17, *p* = 0.878, 95% CI [−0.31, 0.37]). Depression was a significant predictor of PTSS both before including dissociation in the model (B = 0.84, SE = 0.22, *p* < 0.001) and after it (B = 0.82, SE = 0.24, *p* = 0.001). The indirect effect of depression on PTSS through dissociation was not statistically significant (B = 0.01, BootSE = 0.13, 95% percentile bootstrap CI [−0.14, 0.36]; completely standardized indirect effect = 0.01, 95% CI [−0.07, 0.19]), indicating that dissociation did not mediate the association between depression and PTSS. The model explained 25.6% of the variance in dissociation (R^2^ = 0.256), and the predictors jointly explained 35.8% of the variance in PTSS (R^2^ = 0.358). These findings suggest that while depression was related to both dissociation and PTSS, dissociation did not significantly mediate their relationship in this sample, contrary to what was hypothesized, as can be seen in Figure 2.

## 4. Discussion

The present study examined the relationships among depression, dissociation, and post-traumatic stress symptoms (PTSSs) in Emergency Medical Service (EMS) personnel in Israel during the October 2023 war.

In this cross-sectional sample of EMS personnel, higher depressive symptoms, higher DES-II dissociative-experience scores, and higher IES-R post-traumatic stress symptom scores were positively intercorrelated. However, the bootstrapped indirect effect did not support dissociation as a mediator of the association between depression and PTSS. Additionally, the direct association between dissociation and PTSS was not statistically significant after accounting for depression. These findings should be interpreted as symptom-level associations rather than evidence of clinical PTSD, dissociative disorder, or causal pathways.

The observed associations are consistent with previous research demonstrating that first responders are at elevated risk for trauma-related psychological distress because of repeated exposure to fatal accidents, severe injuries, and other highly stressful events [10]. The positive correlations among depression, dissociation, and PTSS also align with findings from both cross-sectional and longitudinal studies showing that depressive symptoms frequently co-occur with dissociative experiences and PTSS in trauma-exposed populations [50,51,52,53].

The significant association between dissociation and PTSS is likewise consistent with systematic reviews identifying dissociation as an important feature of trauma-related psychopathology and, in some individuals, a clinically meaningful component of PTSD [54]. Nevertheless, despite significant associations among all three variables, dissociation did not significantly mediate the relationship between depression and PTSS and was not directly associated with PTSS after accounting for depression. This finding is consistent with and extends a systematic review [55], which concluded that although peritraumatic dissociation is positively associated with PTSD symptoms, there is insufficient evidence to support its role as an independent predictor of PTSD. It suggests that dissociation, although related to both depression and PTSS, may not represent the primary mechanism linking these conditions in EMS personnel.

Several other explanations may also account for the absence of a significant indirect association. First, the relatively modest sample size may have limited statistical power to detect mediation effects, particularly when the indirect association was small. However, the lack of mediation may also reflect the complexity of trauma-related psychopathology. Depression and PTSS are likely connected through multiple interacting cognitive, emotional, and behavioral mechanisms rather than through dissociation alone. Consequently, alternative pathways should be explored in future research [24].

Another consideration relates to the unique composition of Israeli EMS organizations, which rely heavily on both professional staff and volunteers. These groups differ in training, workload, duration of service, and access to organizational support. Volunteers often receive less formal preparation and fewer structured support resources, such as peer-support programs and psychological debriefing, potentially increasing emotional vulnerability [56]. In contrast, professional personnel typically experience greater cumulative occupational exposure, which may influence psychological outcomes through different mechanisms [12]. Although these differences were not examined directly in the present study, they may contribute to variability in psychological responses and warrant further investigation.

Overall, the present findings reinforce previous evidence demonstrating strong relationships among depression, dissociation, and PTSS in trauma-exposed populations, including military veterans and other first responders [57]. At the same time, the absence of a significant mediation effect suggests that the association between depression and PTSS among EMS personnel may be more complex than originally hypothesized in this work. Rather than functioning as a central mediator, dissociation may represent one of several interrelated psychological processes involved in trauma-related psychopathology.

### 4.1. Study Limitations

Several limitations should be considered when interpreting the findings. First, data were collected during the October 2023 war in Israel, an unprecedented period of ongoing national threat and collective trauma. Consequently, participants’ psychological responses may have reflected not only routine occupational exposure inherent to EMS work but also acute distress associated with the broader national emergency. This context may have influenced the expression and interpretation of depression, dissociation, and post-traumatic stress symptoms, limiting the extent to which these findings can be generalized to EMS personnel operating under routine, non-emergency conditions. Accordingly, the present findings should be interpreted as reflecting the psychological functioning of EMS personnel during an active national crisis rather than as representative of their usual occupational experience. Second, participation was voluntary, raising the possibility of selection bias toward more resilient EMS personnel, which may have reduced symptom variability and consequently limited the ability to detect mediation effects. Third, as mentioned in the discussion, professional staff and volunteers were analyzed as a single group, potentially obscuring differences in occupational exposure, coping strategies, and psychological outcomes. Fourth, the cross-sectional design precludes causal inference regarding the relationships among depression, dissociation, and PTSS. Additional limitations include the relatively modest sample size, non-normal data distributions, multiple statistical comparisons, and reliance on self-report measures.

### 4.2. Implications

The findings underscore the need for further research to clarify the distinct relationships between depression, post-traumatic stress symptoms and dissociation in emergency responders. A better understanding of these mechanisms may inform more targeted interventions that address the specific psychological needs of this population.

From a theoretical standpoint, the results contribute to the growing literature on trauma-related psychopathology among first responders, suggesting that while dissociation plays a role in emotional distress, it may not necessarily be the central mechanism linking depression and PTSS. This emphasizes the multifactorial nature of trauma responses in high-risk occupational groups.

### 4.3. Directions for Future Research

Given the study’s limitations, several directions for future research can be proposed. First, combining quantitative assessments with qualitative interviews could provide deeper insight into the lived emotional experiences of EMS personnel and volunteers operating under stress. Second, research should adopt stratified sampling methods or conduct subgroup analyses to distinguish between regular personnel and volunteers to allow better understanding of protective and risk factors such as level of training, perceived organizational support, or motivation to serve.

Finally, a longitudinal design could help determine the directionality of these relationships and clarify the role of dissociation.

## 5. Conclusions

Although depression and dissociation were both strongly associated with PTSS, dissociation did not account for the association between depression and PTSS in this sample of EMS personnel. These findings suggest that the relationship between depression and trauma-related symptoms in emergency responders is likely multifactorial and may involve cognitive, emotional, and behavioral processes beyond dissociation alone. Given the repeated occupational exposure to trauma experienced by EMS personnel, identifying these mechanisms may contribute to a better understanding of trauma-related psychopathology and inform more targeted screening, prevention, and intervention strategies aimed at promoting the mental health and resilience of emergency responders.

## Figures and Tables

**Table 1:** Demographic characteristics of the study sample.

Variable	Category	*n* (%)	Mean ± SD	Median	Range
Gender	Male	35 (58.3)			
	Female	25 (41.7)			
District	Jerusalem	25 (41.6)			
	North	14 (23.3)			
	Central	15 (25.0)			
	South	6 (10.0)			
Traumatic experience	Yes	38 (61.6)			
	No	22 (38.3)			
Age		60 (100)	29.18 ± 12.96	25.5	18–67
Seniority in EMS		59 (98.3)	4.94 ± 5.2	3.5	0.3–30
Number of shifts per month		58 (96.6)	10.7 ± 8.6	8	1–28

Note. Continuous variables are presented as mean ± SD, median, and range; categorical variables are presented as *n* (%). EMS, emergency medical services.

**Table 2:** Study variables.

**(A). Severity Categories**			
**Variable**	**Category**	***n* (%)**	
IES-R	Normal	58.3	
	Mild	11.66	
	Moderate	5.0	
	Severe	25.0	
DASS-21	Normal	60.0	
Depression			
Subscale			
	Mild	13.33	
	Moderate	15.0	
	Severe	5.0	
	Extremely severe	6.66	
DES	Up to 30%	70.0	
	Over 30%	30.0	
**(B). Descriptive Statistics**			
**Variable**	**Mean ± SD**	**Median**	**Range**
IES-R	24.96 ± 19.53	21.0	1–72
DASS-21	8.50 ± 10.01	4.0	0–42
Depression			
Subscale			
DES	25.97 ± 14.08	22.14	6.4–82.5

Note. Categorical variables are presented as percentages; continuous variables are presented as mean ± SD, median, and range. Abbreviations: IES-R, Impact of Event Scale–Revised; DASS-21, Depression, Anxiety, and Stress Scale; DES, Dissociative Experiences Scale.

**Table 3:** The relationship between background variables and PTSS, depression, and dissociation.

Variables		*n*	Mean	SD	Median	*p*-Value
IES-R						
Gender	Male	35	22.71	18.86	21.0	
	Female	25	28.12	18.48	23.0	0.274
District	Jerusalem	25	20.44	14.16	18.0	
	North	14	32.21	22.51	29.0	
	Central	15	24.4	22.78	19.0	
	South	6	28.3	12.17	26.0	0.304
Traumatic experience	Yes	38	25.6	18.58	21.5	
	No	22	23.86	19.41	21.0	0.368
Exposure to potentially traumatic scene	No	4	11.25	6.7	9.0	
	Witnessing death of a patient	18	29.38	22.58	23.5	
	Treating a severely injured patient	21	26.85	17.21	25.0	
	Providing medical care in a gruesome motor vehicle accident	17	21.2	16.9	14.0	0.231
DASS-21						
Gender	Male	35	6.45	9.2	2.0	
	Female	25	11.36	10.57	10.0	0.034 *
	Jerusalem	25	7.12	10.05	2.0	
	North	14	11.71	11.41	12.0	
	Central	15	8.0	8.0	7.96	
	South	6	8.0	11.93	4.0	0.496
Traumatic experience	Yes	38	10.31	10.82	7.0	
	No	22	5.36	7.69	1.0	0.022 *
Exposure to potentially traumatic scene	No	4	0.0	0.0	0.0	
	Witnessing death of a patient	18	8.9	12.2	3.0	
	Treating a severely injured patient	21	10.47	10.42	8.0	
	Providing medical care in a gruesome motor vehicle accident	17	7.6	7.14	4.0	0.066
DES						
Gender	Male	35	25.1	15.25	21.07	
	Female	25	27.2	12.46	24.64	0.561
	Jerusalem	25	21.85	9.34	20.0	
	North	14	34.15	19.54	29.1	
	Central	15	25.16	14.8	19.64	
	South	6	26.07	6.28	25.89	0.141
Traumatic experience	Yes	38	27.31	12.4	23.75	
	No	22	23.66	16.6	19.82	0.189
Exposure to potentially traumatic scene	No	4	16.16	9.42	14.82	
	Witnessing death of a patient	18	25.3	14.3	20.35	
	Treating a severely injured patient	21	26.2	11.8	27.14	
	Providing medical care in a gruesome motor vehicle accident	17	28.7	17.0	22.5	0.344

* *p* < 0.05 (two-tailed). Abbreviations: IES-R, Impact of Event Scale–Revised; DASS-21, Depression, Anxiety, and Stress Scale; DES, Dissociative Experiences Scale.

**Table 4:** Spearman correlations of PTSS, depression, dissociation, and corresponding symptoms.

	2	3	4	5	6	7	8	9	10	11
1. PTSS	0.913 **	0.937 **	0.910 **	0.536 **	0.451 **	0.401 **	0.561 **	0.519 **	0.592 **	0.622 **
2. Avoidance		0.755 **	0.775 **	0.508 **	0.430 **	0.324	0.518 **	0.498 **	0.568 **	0.574 **
3. Intrusion			0.818 **	0.485 **	0.411 **	0.379 **	0.506 **	0.471 **	0.570 **	0.576 **
4. Hyperarousal				0.431 **	0.415 **	0.380 **	0.452 **	0.435 **	0.532 **	0.613 **
5. Dissociation					0.764 **	0.835 **	0.875 **	0.512 **	0.520 **	0.483 **
6. Amnesia						0.615 **	0.549 **	0.197	0.293	0.302
7. Depersonalization							0.692 **	0.516 **	0.554 **	0.483 **
8. Absorption								0.520 **	0.518 **	0.465 **
9. Depression									0.652 **	0.821 **
10. Anxiety										0.714 **
11. Stress										

** Correlation is significant at the 0.01 level (2-tailed).

**Table 5:** Mediation analysis with dissociation as a mediator between depression and PTSS.

Path	Predictor → Outcome	β	*B*	*SE*	*t*	*p*	95% CI
a	Depression → Dissociation	0.38	0.53	0.18	2.97	0.004	[0.17, 0.90]
b	Dissociation → PTSS	0.02	0.03	0.17	0.15	0.878	[−0.31, 0.37]
c	Depression → PTSS (total)	0.45	0.84	0.22	3.76	<0.001	[0.39, 1.28]
c′	Depression → PTSS (direct)	0.44	0.82	0.24	3.40	0.001	[0.34, 1.31]
a × b	Indirect via Dissociation	0.01	0.01	0.13	–	–	[−0.14, 0.36] ^a^

Note. a = path from the independent variable to the mediator; b = path from the mediator to the dependent variable controlling for the independent variable; c = total effect of the independent variable on the dependent variable; c′ = direct effect controlling for the mediator; a × b = indirect (mediated) effect. *B* = unstandardized coefficient; β = standardized coefficient. DES-II scored on its conventional 0–100 metric. All paths adjusted for gender, age, years of EMS experience, and external stress exposure. ^a^ Percentile bootstrap confidence interval (5000 samples, seed = 12,345); *SE* is the bootstrap standard error. CIs for paths a, b, c, and c′ refer to the unstandardized coefficients (*B*).

## Data Availability

The raw data supporting the conclusions of this article will be made available by the authors upon request.

## References

[1] Cloitre M., Hyland P., Bisson J.I., Brewin C.R., Roberts N.P., Karatzias T., Shevlin M. (2019). ICD-11 Posttraumatic Stress Disorder and Complex Posttraumatic Stress Disorder in the United States: A Population-Based Study. J. Trauma. Stress.

[2] American Psychiatric Association (2022). Diagnostic and Statistical Manual of Mental Disorders.

[3] Kessler R.C., Aguilar-Gaxiola S., Alonso J., Benjet C., Bromet E.J., Cardoso G., Degenhardt L., de Girolamo G., Dinolova R.V., Ferry F. (2017). Trauma and PTSD in the WHO World Mental Health Surveys. Eur. J. Psychotraumatol..

[4] Bisson J.I., Cosgrove S., Lewis C., Roberts N.P. (2015). Post-traumatic stress disorder. BMJ.

[5] Stanley I.H., Hom M.A., Joiner T.E. (2016). A systematic review of suicidal thoughts and behaviors among police officers, firefighters, EMTs, and paramedics. Clin. Psychol. Rev..

[6] Kerai S.M., Khan U.R., Islam M., Asad N., Razzak J., Pasha O. (2017). Post-traumatic stress disorder and its predictors in emergency medical service personnel: A cross-sectional study from Karachi, Pakistan. BMC Emerg. Med..

[7] Hoell A., Kourmpeli E., Dressing H. (2023). Work-related posttraumatic stress disorder in paramedics in comparison to data from the general population of working age: A systematic review and meta-analysis. Front. Public Health.

[8] Ntatamala I., Adams S. (2022). The correlates of post-traumatic stress disorder in ambulance personnel and barriers faced in accessing care for work-related stress. Int. J. Environ. Res. Public Health.

[9] Greinacher A., Derezza-Greeven C., Herzog W., Nikendei C. (2019). Secondary traumatization in first responders: A systematic review. Eur. J. Psychotraumatol..

[10] Møller S., Hansen A.S., Pihl-Thingvad J., Elklit A., Hansen N.B. (2023). Critical Incidents in Police Work: What Incidents Stay with Danish Police Officers?. J. Police Crim. Psychol..

[11] Rimon A., Shelef L. (2025). Moral injury among medical personnel and first responders across different healthcare and emergency response settings: A narrative review. Int. J. Environ. Res. Public Health.

[12] Berger W., Coutinho E.S.F., Figueira I., Marques-Portella C., Luz M.P., Neylan T.C., Marmar C.R., Mendlowicz M.V. (2012). Rescuers at risk: A systematic review and meta-regression analysis of the worldwide current prevalence and correlates of PTSD in rescue workers. Soc. Psychiatry Psychiatr. Epidemiol..

[13] Kolassa I.-T., Ertl V., Eckart C., Kolassa S., Onyut L.P., Elbert T. (2010). Spontaneous remission from PTSD depends on the number of traumatic event types experienced. Psychol. Trauma.

[14] Donnelly E. (2012). Work-related stress and posttraumatic stress in emergency medical services. Prehosp. Emerg. Care.

[15] Rytwinski N.K., Scur M.D., Feeny N.C., Youngstrom E.A. (2013). The co-occurrence of major depressive disorder among individuals with posttraumatic stress disorder: A meta-analysis. J. Trauma. Stress.

[16] Claxton J., Vibhakar V., Allen L., Finn J., Gee B., Meiser-Stedman R. (2021). Risk factors for depression in trauma-exposed children and adolescents: A systematic review and meta-analysis. J. Affect. Disord. Rep..

[17] Memarzia J., Lofthouse K., Dalgleish T., Boyle A., McKinnon A., Dixon C., Smith P., Meiser-Stedman R. (2024). Predictive models of post-traumatic stress disorder, complex post-traumatic stress disorder, depression, and anxiety in children and adolescents following a single-event trauma. Psychol. Med..

[18] Kessler R.C., Bromet E.J. (2013). The epidemiology of depression across cultures. Annu. Rev. Public Health.

[19] Otte C., Gold S.M., Penninx B.W., Pariante C.M., Etkin A., Fava M., Mohr D.C., Schatzberg A.F. (2016). Major depressive disorder. Nat. Rev. Dis. Primers.

[20] Flory J.D., Yehuda R. (2015). Comorbidity between post-traumatic stress disorder and major depressive disorder: Alternative explanations and treatment considerations. Dialogues Clin. Neurosci..

[21] Post L.M., Youngstrom E., Connell A.M., Zoellner L.A., Feeny N.C. (2021). Transdiagnostic emotion regulation processes explain how emotion-related factors affect co-occurring PTSD and MDD in relation to trauma. J. Anxiety Disord..

[22] Post L.M., Zoellner L.A., Youngstrom E., Feeny N.C. (2011). Understanding the relationship between co-occurring PTSD and MDD: Symptom severity and affect. J. Anxiety Disord..

[23] Payne A. (2018). Intrusive Memories in Depression and Posttraumatic Stress Disorder. Doctoral Dissertation.

[24] Shelef L., Spira N., Bechor U., Rotschield J., Shadach E. (2025). The roles of dissociation and depression in PTSD among soldiers exposed to combat. Int. J. Environ. Res. Public Health.

[25] Spiegel D., Loewenstein R.J., Lewis-Fernández R., Şar V., Simeon D., Vermetten E., Cardeña E., Dell P.F. (2011). Dissociative disorders in DSM-5. Depress. Anxiety.

[26] van der Hart O. (2021). Trauma-Related Dissociation: An Analysis of Two Conflicting Models. Eur. J. Trauma Dissociation.

[27] Lanius R.A., Vermetten E., Loewenstein R.J., Brand B., Schmahl C., Bremner J.D., Spiegel D. (2010). Emotion modulation in PTSD: Clinical and neurobiological evidence for a dissociative subtype. Am. J. Psychiatry.

[28] Patel H., O’Connor C., Andrews K., Amlung M., Lanius R., McKinnon M.C. (2022). Dissociative symptomatology mediates the relation between posttraumatic stress disorder severity and alcohol-related problems. Alcohol. Clin. Exp. Res..

[29] Spiegel D., Lewis-Fernández R., Lanius R., Vermetten E., Simeon D., Friedman M. (2013). Dissociative disorders in DSM-5. Annu. Rev. Clin. Psychol..

[30] Loewenstein R.J. (2018). Dissociation debates: Everything you know is wrong. Dialogues Clin. Neurosci..

[31] Şar V. (2015). Dissociative depression is resistant to treatment-as-usual. J. Psychol. Clin. Psychiatry.

[32] Fung H.W., Chien W.T., Lam S.K.K., Ross C.A. (2022). Prevalence and correlates of dissociative symptoms among people with depression. J. Psychiatr. Res..

[33] McCanlies E.C., Sarkisian K., Andrew M.E., Burchfiel C.M., Violanti J.M. (2017). Association of peritraumatic dissociation with symptoms of depression and posttraumatic stress disorder. Psychol. Trauma.

[34] Minnie L., Goodman S., Wallis L. (2015). Exposure to daily trauma: The experiences and coping mechanisms of emergency medical personnel. A cross-sectional study. Afr. J. Emerg. Med..

[35] Weiss D.S., Marmar C.R., Wilson J.P., Keane T.M. (1997). The Impact of Event Scale-Revised. Assessing Psychological Trauma and PTSD: A Practitioner’s Handbook.

[36] Somer E., Ruvio A., Soref E., Sever I. (2005). Terrorism, distress and coping: High versus low impact regions and direct versus indirect civilian exposure. Anxiety Stress Coping.

[37] Horowitz M., Wilner N., Alvarez W. (1979). Impact of Event Scale: A measure of subjective stress. Psychosom. Med..

[38] Sveen J., Low A., Dyster-Aas J., Ekselius L., Willebrand M., Gerdin B. (2010). Validation of a Swedish version of the Impact of Event Scale-Revised (IES-R) in patients with burns. J. Anxiety Disord..

[39] Willebrand M., Wikehult B., Ekselius L. (2004). Acceptance of a trauma-focused survey: Do personality and health matter?. Gen. Hosp. Psychiatry.

[40] Lovibond P.F., Lovibond S.H. (1995). Manual for the Depression Anxiety Stress Scales.

[41] Lurie J. DASS-21 (Hebrew Translation).

[42] Henry J.D., Crawford J.R. (2005). The short-form version of the Depression Anxiety Stress Scales (DASS-21): Construct validity and normative data in a large non-clinical sample. Br. J. Clin. Psychol..

[43] Bernstein E.M., Putnam F.W. (1986). Development, reliability, and validity of a dissociation scale. J. Nerv. Ment. Dis..

[44] Carlson E.B., Putnam F.W. (1993). An update on the Dissociative Experiences Scale. Dissociation.

[45] Somer E., Dolgin M., Saadon M. (2001). Validation of the Hebrew version of the Dissociative Experiences Scale (H-DES) in Israel. J. Trauma Dissociation.

[46] Frischholz E.J., Braun B.G., Sachs R.G., Hopkins L., Shaeffer D.M., Lewis J., Leavitt F., Pasquotto J.N., Schwartz D.R. (1990). The Dissociative Experiences Scale: Further replication and validation. Dissociation.

[47] Lyssenko L., Schmahl C., Bockhacker L., Vonderlin R., Bohus M., Kleindienst N. (2018). Dissociation in psychiatric disorders: A meta-analysis of studies using the Dissociative Experiences Scale. Am. J. Psychiatry.

[48] Hayes A.F., Montoya A.K., Rockwood N.J. (2017). The analysis of mechanisms and their contingencies: PROCESS versus structural equation modeling. Australas. Mark. J..

[49] Benjamini Y., Hochberg Y. (1995). Controlling the false discovery rate: A practical and powerful approach to multiple testing. J. R. Stat. Soc. Ser. B Methodol..

[50] Fung H.W., Chau A.K.C., Hung S.L., Lam S.K.K., Chien W.T., Lee V.W.P. (2023). Persistence and clinical consequences of post-traumatic and dissociative symptoms in people with depressive symptoms: A one-year follow-up study. Eur. J. Psychotraumatol..

[51] Fung H.W., Cheung C.T.Y. (2024). The bidirectional relationship between depression and dissociation: A longitudinal investigation. Asian J. Psychiatry.

[52] Černis E., Evans R., Ehlers A., Freeman D. (2021). Dissociation in relation to other mental health conditions: An exploration using network analysis. J. Psychiatr. Res..

[53] Moulds M.L., Bisby M.A., Wild J., Bryant R.A. (2020). Rumination in posttraumatic stress disorder: A systematic review. Clin. Psychol. Rev..

[54] Atchley R., Bedford C. (2021). Dissociative symptoms in posttraumatic stress disorder: A systematic review. J. Trauma Dissociation.

[55] van der Velden P.G., Wittmann L. (2008). The Independent Predictive Value of Peritraumatic Dissociation for PTSD Symptomatology after Type I Trauma: A Systematic Review of Prospective Studies. Clin. Psychol. Rev..

[56] Jaffe E., Sasson U., Knobler H., Aviel E., Goldberg A. (2012). Volunteers and the risk of posttraumatic stress disorder. Nonprofit Manag. Leadersh..

[57] Wisco B.E., Nomamiukor F.O., Marx B.P., Krystal J.H., Southwick S.M., Pietrzak R.H. (2022). Posttraumatic stress disorder in U.S. military veterans: Results from the 2019–2020 National Health and Resilience in Veterans Study. J. Clin. Psychiatry.

